# Analyzing the Influence of Modic Changes on Patients with Lower Back Pain Undergoing Conservative Treatment

**DOI:** 10.1155/2019/8185316

**Published:** 2019-03-19

**Authors:** Yufeng Chen, Huilin Yang, Lianfang Zhang, Yue Wang, Jun Zou

**Affiliations:** ^1^Department of Orthopaedic Surgery, The First Affiliated Hospital of Soochow University, Suzhou, Jiangsu 215006, China; ^2^Department of Orthopaedic Surgery, The First Affiliated Hospital of Zhejiang University, Hangzhou, Zhejiang 310003, China

## Abstract

**Objective:**

This study aimed to investigate if the presence of Modic changes (MCs) was correlated with lower back pain (LBP) and LBP-related disability in patients who underwent nonsurgical treatment.

**Methods:**

In this study, 129 patients who experienced consecutive LBP and underwent lumbar spine magnetic resonance imaging in our institute were divided into three groups according to the presence or type of MCs. The Oswestry Disability Index (ODI) and visual analog scale (VAS) were used to assess the outcomes of the treatment.

**Results:**

Based on the achieved results, there was no significant difference between three groups before treatment (*P* > 0.05). Three months after undergoing nonsurgical treatment, the rates of improved ODI and VAS scores were statistically significantly different (*P*=0.014, 0.023). After an additional 3 months of treatment, in patients with Modic type I changes, the symptoms significantly improved in comparison with those 3 months prior (*P*=0.037, 0.026), while that improvement did not occur in patients with Modic type II changes (*P* > 0.05).

**Conclusions:**

The existence of MCs affects the outcomes of nonsurgical treatment in patients with LBP. However, symptoms can be improved after an additional round of treatment for Modic type I changes, while this is not confirmed for Modic type II changes.

## 1. Introduction

Lower back pain (LBP) is a common health problem, and an estimated 80% of adults have experienced LBP at least once during their lifetime [1]. LBP nowadays seriously threatens Western societies in terms of the years lived with disability (YLDs) [2]. A nonsurgical treatment technique has shown significant efficacy in treating LBP. It is noteworthy that around 11% of patients with LBP require an additional surgery [3]. However, the main challenge for nonsurgical treatment is to identify the most appropriate intervention [4]. In addition, 85% of patients with isolated LBP cannot be precisely diagnosed with pathoanatomical methods [5]. The unknown etiologies may result in poor outcomes of nonsurgical treatment in some patients as well.

Modic changes (MCs) are a common phenomenon observed using magnetic resonance imaging (MRI) in spinal degenerative diseases and are greatly associated with LBP [6]. MCs are classified based on T1-weighted (T1W), T2-weighted (T2W), and T2W with fat suppression images in MRI [6, 7]. Modic type I changes are basically regarded as bone marrow edema and inflammation. In addition, Modic type II changes represent fatty degeneration of the bone marrow. MCs are associated with age, gender, body weight, smoking, previous spinal cord injuries, and physical workload [8]. Some researchers have previously reported a close relationship between MCs and LBP, particularly for type I changes [9–12]. According to our knowledge, the influence of MCs on patients with LBP has not been deeply studied yet. Hence, the present study retrospectively analyzed if the presence of MCs was correlated with LBP and LBP-related disability in patients who underwent nonsurgical treatment.

## 2. Materials and Methods

Herein, 129 patients who were admitted to the Department of Orthopaedic Surgery in the First Affiliated Hospital of Soochow University between January 2013 and December 2015 were enrolled. This study was approved by the Ethics Committee of the first affiliated hospital of Soochow University and was in accordance with the Helsinki Declaration. Written informed consent was obtained from every participant. Several criteria were adopted, involving age with a range of 20–70 years, LBP experienced for 3 to 12 months, without radicular leg pain, and no history of formal treatment. The exclusion criteria were mixed MCs, a history of abdominal/pelvic surgery, as well as a specific spinal disease (e.g., scoliosis, spondylolisthesis, infection, and tumor).

Two experienced surgeons evaluated the images according to the criteria presented by Modic [6] for the presence of subchondral signal abnormalities. All patients used the same machine, and both T1W and T2W scanned 12 images from left to right and two consecutive images with abnormal signal changes are considered to have MCs. The overall interobserver agreement was excellent with a *κ* value of 0.85.

Patients were divided into three groups. Group A consisted of 50 patients without MCs, involving 22 men and 28 women with the age range of 25–62 years (a mean age of 40.5 years). Group B involved 31 patients with Modic type I changes, including 13 men and 18 women with the age range of 21–65 years (a mean age of 41.6 years). Group C consisted of 48 patients with Modic type II changes, involving 21 men and 27 women with the age range of 24–67 years (a mean age of 44.5 years), which are listed in Table 1.

### 2.1. Assessment of Images

**Figure 1:**
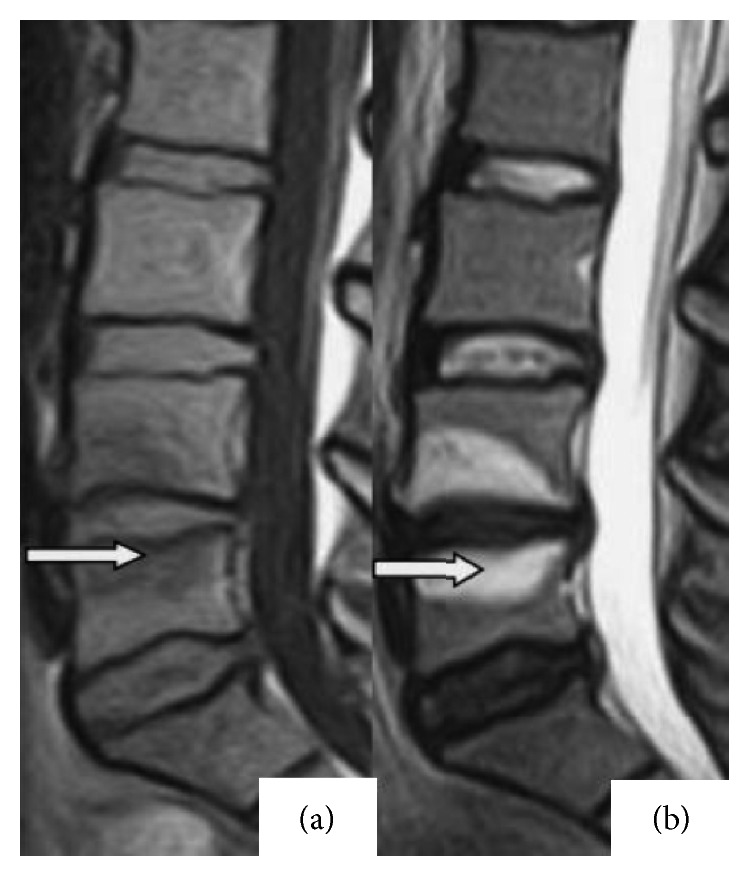
Illustration of Modic type I changes. (a) Hypointense changes on T1-weighted images at the L4 vertebral lower endplate and L5 vertebral body upper endplate. (b) Hyperintense changes on T2-weighted images at the L4 vertebral lower endplate and L5 vertebral upper endplate.

**Figure 2:**
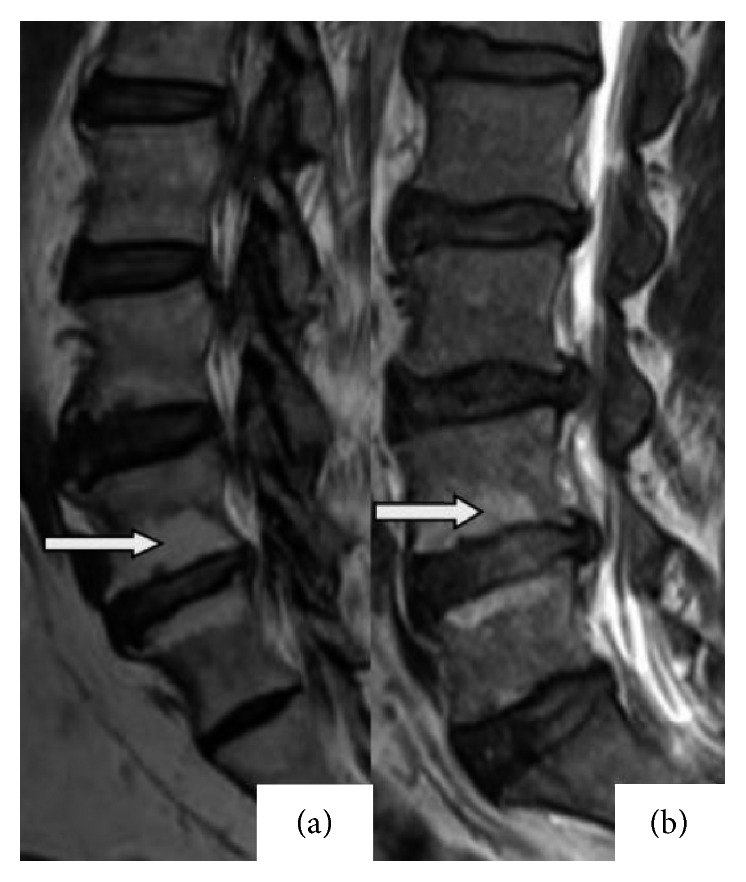
Illustration of Modic type II changes. (a) Hyperintense changes on T1-weighted images at the L4 vertebral lower endplate and L5 vertebral body upper endplate. (b) Hyperintense changes on T2-weighted images at the L4 vertebral lower endplate and L5 vertebral upper endplate.

Modic type I changes reflect hypointense and hyperintense signals on T1W and T2W images, respectively, while Modic type II changes represent hyperintense signals on T1W images and isointense or slightly hyperintense signals on T2W images, as illustrated in Figures 1 and 2.

### 2.2. Assessment of Symptoms

Two experienced nurses collected and completed the questionnaires according to patients' symptoms. For this purpose, the Oswestry Disability Index (ODI), derived from the Oswestry Low Back Pain Questionnaire, and visual analog scale (VAS) were utilized to assess the severity of symptoms, and rates of improved ODI and VAS scores were used to assess the efficacy of treatment. For this purpose, the rate of improved ODI scores was calculated as ((prior ODI − follow-up ODI)/prior ODI) × 100%, and the rate for improved VAS scores was calculated as ((prior VAS − follow-up VAS)/prior VAS) × 100%.

### 2.3. Nonsurgical Treatment

All patients underwent nonsurgical treatment for 6 months (two courses) involving the McKenzie method and pharmacological therapy. Regarding the McKenzie method, the treatment procedure occurs in only one position, consisting of a number of stages which are as follows: lie on a hard bed in the prone position, place head and leg at angles up to 20 degrees each, place hands on back, hold this position for 15 seconds, then release slowly, rest for 5 seconds, and repeat the action 30 times twice per day. Some small adjustments were undertaken according to each patient's characteristics. Using pharmacological therapy, nonsteroidal anti-inflammatory drugs (NSAIDs) and muscle relaxants were administered once per day for two weeks. Patients were advised to continue consuming the drugs if pain was unrelieved, but not for more than four weeks. Next, patients were asked to select a hard bed, in lieu of a soft bed, and lie on it as much as possible for 3 months; then, the bending and sedentary time was decreased, as well as the workload to avoid increasing the waist load. A traditional Chinese medicine (TCM) therapeutic massage was performed, which relaxed whole muscles. Afterward, an experienced therapist repeatedly and gently massaged patients' back muscles for 30 minutes, once a week for 3 months.

### 2.4. Statistical Analysis

The data were presented as means and standard deviations. A one-way analysis of variance (ANOVA) was used to compare the basic characteristics of VAS and ODI scores, as well as rates of improved ODI and VAS scores among the three presented groups. The chi-squared test was used to compare numbers of smokers and heavy workers or gender distribution between the groups. A paired sample *t*-test was used to compare the ODI and VAS scores at different time points in each group. *P* values less than 0.05 were considered statistically significant. All data were analyzed using SPSS software version 22.0 for Windows (SPSS Inc., IL, USA).

## 3. Results

There were no statistically significant differences in gender, body weight, workload, smoking, or involved discs between the three groups (Table 1). Before treatment, the ODI scores for no MC, MC1, and MC2 were 22.7 ± 4.6, 22.0 ± 5.2, and 22.7 ± 5.1, respectively, and those for VAS scores were 6.3 ± 1.6, 6.4 ± 1.8, and 6.3 ± 2.2, respectively. No significant difference was found between the three groups (*P* > 0.05), as mentioned in Table 2. Three months after undergoing nonsurgical treatment, the rates of improved ODI scores were 60.8%, 57.7%, and 48.0%, respectively, and those for improved VAS scores were 61.9%, 54.7%, and 46.0%, respectively. There was a significant difference between the three groups (*P* < 0.05). An additional 3 months after undergoing the treatment, in the MC1 group, again the rates of improved ODI and VAS scores were 16.1% and 13.8%, respectively, which were significantly higher than those 3 months priorly. However, no significant improvement was found in the MC1 group, as demonstrated in Tables 3 and 4.

## 4. Discussion

It has been reported that endplate changes are associated with LBP, and our previous research revealed that four types of endplate lesions (Schmorl's nodes, fracture, erosion, and calcification) were associated with disc degeneration as well as LBP [13, 14]. However, whether they have an influence on nonsurgical treatment remains unknown.

The presented nonsurgical treatment involves McKenzie exercises (extension in lying), pharmacological therapy, bed rest, change in lifestyle, and TCM therapeutic massage. McKenzie exercises can increase the strength of lumbar muscles, and several researchers have previously assessed the role and activation patterns of the trunk musculature as they correlate with the concept of spinal stability [15]. NSAIDs function through various degrees of reversible blockade of cyclooxygenase isoenzymes (COX-1 and COX-2), thus blocking the inflammatory cascade of arachidonic acid to prostaglandins, mediating inflammation as well as sensitizing peripheral nociceptors. Muscle relaxants generally act through inhibiting central polysynaptic neuronal events, indirectly acting on skeletal muscle [16]. Bed rest and massages could reduce waist load, which relaxes back muscles and relieves pain as well.

Some researchers have reported that cases of LBP with MCs are mainly related to inflammation [17–19]. Inflammatory factors, e.g., interleukin 6, interleukin 8, prostaglandin E2, and tumor necrosis factor alpha, cause pain after stimulating nerve endings. Crock [17] suggested that upregulation of inflammatory mediators in the nucleus pulpous could be associated with a local inflammation response. Zhang et al. [18] found that nucleus pulposus could produce a series of inflammatory factors and transmit them to vertebrae through the fissures in endplates. Ohtori et al. [19] reported that the expression of inflammatory factors in endplates from patients with Modic type I changes was significantly higher than that in endplates from patients with Modic type II changes. Therefore, NSAIDs might have a sensitive efficacy to LBP because of the control of pain induced by inflammation. This can be justified by the finding that the improvement rate of pain 3 months after the treatment in group B (54.7%) was remarkably higher than that in group C (46.0%), reflecting that Modic type I changes demonstrated a superior outcome than Modic type II changes.

Toyone et al. [20] studied 74 patients with MCs and found that 70.3% (26/37) of patients with Modic type I changes had LBP, while only 16.2% (6/37) of patients with Modic type II changes had LBP. They reported that type I changes were correlated with segmental instability and LBP, while type II changes were more common in patients with stable degenerative disc disease. However, the relationship between MCs and segmental instability is mostly supported by indirect evidence of the efficacy of lumbar fusion surgery [21]. Kulig et al. [22] investigated 45 patients with LBP and 20 patients without LBP and determined that LBP has a strong correlation with segmental instability. In the present study, the rate of improved ODI enhanced to 16.1% after an additional round of treatment in group B, while that rate was only 4.2% in group C. It is apparent that a disability symptom can be improved after an additional round of treatment for Modic type I changes; however, that is not feasible for Modic type II changes. McKenzie extension exercises could increase muscle strength, resulting in an increase in lumbar segmental stability, as the exercise itself and the strength of muscle demonstrate a long process; as for patients with type I changes, an additional round of treatment could facilitate satisfactory results.

Modic type I and type II changes represent different stages of the same pathological process [9]. Mitra et al. [23] investigated 44 patients with type I changes with a follow-up that lasted for 12–72 months. They found that 37.5% (18/48) of patients fully converted to type II changes and 14.6% (7/48) of patients partially converted to type II changes. They demonstrated that type I changes are an acute phase and eventually can be transformed into other types, and a positive correlation was found in the evolution of type I changes to type II changes, as well as symptom improvement. Hutton et al. [24] reported a similar conclusion with a study of 36 endplate cases. In the current study, the rate of improved VAS enhanced to 13.8% after an additional round of treatment in group B; however, the rate was only 5.9% in group C, reflecting that pain symptoms can be relieved after an additional round for type I changes, while no significant intensity was found for type II changes. As a result, over time, Modic type I changes would have superior intensity of pain than Modic type II changes.

Modic type III changes were not included in this study, as the population of study samples was small, and this is a preliminary study with a short follow-up, requiring further investigation.

## 5. Conclusions

In summary, the existence of MCs affects the outcomes of nonsurgical treatment in patients with LBP. In addition, the outstanding role of a formal nonsurgical treatment and the importance of confidence were revealed. Moreover, symptoms can be improved after an additional round of treatment through type I changes.

## Figures and Tables

**Table 1:** Fundamental characteristics of the three groups before treatment.

	Group A	Group B	Group C
Number	50	31	48
Male	22 (44%)	13 (42%)	21 (44%)
Female	28 (56%)	18 (58%)	27 (56%)
Average age (range)	40.5 (25–62)	41.6 (21–65)	44.5 (24–67)
BMI	20.4 ± 1.0	20.1 ± 0.9	20.5 ± 0.9
Smoking	13 (26.0%)	8 (25.8%)	14 (29.2%)
Heavy work	29 (58%)	18 (58.1%)	28 (58.3%)
Involved disc			
L3–4		3	3
L4–5		13	20
L5–S1		15	25

**Table 2:** Scores of ODI and VAS for the three groups before treatment.

	Group A	Group B	Group C
Number	50	31	48
Male : female	22 : 28	13 : 18	21 : 27
ODI	22.7 ± 4.6	22.0 ± 5.2	22.7 ± 5.1
VAS	6.3 ± 1.6	6.4 ± 1.8	6.3 ± 2.2

**Table 3:** Scores of ODI and VAS, as well as corresponding improved rates for the three groups 3 months after treatment.

	Group A	Group B	Group C	*P*
Number	50	31	48	
ODI	8.9 ± 2.1	9.3 ± 2.4	11.8 ± 2.9	
ODI improvement rate (%)	60.8	57.7	48.0	0.014^*∗*^
VAS	2.4 ± 0.8	2.9 ± 0.8	3.4 ± 1.2	
VAS improvement rate (%)	61.9	54.7	46.0	0.023^*∗*^

^*∗*^Statistically significant.

**Table 4:** Scores of ODI and VAS and corresponding improved rates for the three groups 6 months after treatment, as well as comparing with the results in Table 3.

	Group A	Group B	Group C
Number	50	31	48
ODI	8.5 ± 2.0	7.8 ± 2.8	11.3 ± 2.6
ODI improvement rate (%)	4.5	16.1	4.2
*P*	0.130	0.037^*∗*^	0.178
VAS	2.3 ± 0.8	2.5 ± 1.2	3.2 ± 0.9
VAS improvement rate (%)	4.2	13.8	5.9
*P*	0.097	0.026^*∗*^	0.082

^*∗*^Statistically significant.

## Data Availability

The data used to support the findings of this study are available from the corresponding author upon request.

## References

[1] Andersson G. B. (1999). Epidemiological features of chronic low-back pain. *The Lancet*.

[2] Murray C. J. L., Lopez A. D. (2013). Measuring the global burden of disease. *New England Journal of Medicine*.

[3] Jonsson E., Olafsson G., Fritzell P., Hägg O., Borgström F. (2017). A profile of low back pain: treatment and costs associated with patients referred to orthopedic specialists in Sweden. *Spine*.

[4] Maluf K. S., Sahrmann S. A., Van Dillen L. R. (2000). Use of a classification system to guide nonsurgical management of a patient with chronic low back pain. *Physical Therapy*.

[5] Deyo R. A., Weinstein J. N. (2001). Low back pain. *New England Journal of Medicine*.

[6] Modic M. T., Steinberg P. M., Ross J. S., Masaryk T. J., Carter J. R. (1988). Degenerative disk disease: assessment of changes in vertebral body marrow with MR imaging. *Radiology*.

[7] Modic M. T., Masaryk T. J., Ross J. S., Carter J. R. (1988). Imaging of degenerative disk disease. *Radiology*.

[8] Mok F. P. S., Samartzis D., Karppinen J., Fong D. Y. T., Luk K. D. K., Cheung K. M. C. (2016). Modic changes of the lumbar spine: prevalence, risk factors, and association with disc degeneration and low back pain in a large-scale population-based cohort. *The Spine Journal*.

[9] Braithwaite I., White J., Saifuddin A., Renton P., Taylor B. A. (1998). Vertebral end-plate (Modic) changes on lumbar spine MRI: correlation with pain reproduction at lumbar discography. *European Spine Journal*.

[10] Jensen T. S., Karppinen J., Sorensen J. S., Niinimäki J., Leboeuf-Yde C. (2008). Vertebral endplate signal changes (Modic change): a systematic literature review of prevalence and association with non-specific low back pain. *European Spine Journal*.

[11] Kjaer P., Leboeuf-Yde C., Korsholm L., Sorensen J. S., Bendix T. (2005). Magnetic resonance imaging and low back pain in adults: a diagnostic imaging study of 40-year-old men and women. *Spine*.

[12] Thompson K. J., Dagher A. P., Eckel T. S., Clark M., Reinig J. W. (2009). Modic changes on MR images as studied with provocative diskography: clinical relevance—a retrospective study of 2457 disks. *Radiology*.

[13] Wang Y., Videman T., Battié M. C. (2012). Lumbar vertebral endplate lesions: prevalence, classification and association with age. *Spine*.

[14] Wang Y., Videman T., Battié M. C. (2012). ISSLS prize winner: lumbar vertebral endplate lesions associations with disc degeneration and back pain history. *Spine*.

[15] Standaert C. J., Weinstein S. M., Rumpeltes J. (2008). Evidence-informed management of chronic low back pain with lumbar stabilization exercises. *The Spine Journal*.

[16] Malanga G., Wolff E. (2008). Evidence-informed management of chronic low back pain with nonsteroidal anti-inflammatory drugs, muscle relaxants, and simple analgesics. *The Spine Journal*.

[17] Crock H. V. (1986). The presidential address: ISSLS: internal disc disruption: a challenge to disc prolapse fifty years on. *Spine*.

[18] Zhang Y.-H., Zhao C.-Q., Jiang L.-S., Chen X.-D., Dai L.-Y. (2008). Modic changes: a systematic review of the literature. *European Spine Journal*.

[19] Ohtori S., Inoue G., Ito T. (2006). Tumor necrosis factor-immunoreactive cells and PGP 9.5-immunoreactive nerve fibers in vertebral endplates of patients with discogenic low back pain and Modic type 1 or type 2 changes on MRI. *Spine*.

[20] Toyone T., Takahashi K., Kitahara H., Yamagata M., Murakami M., Moriya H. (1994). Vertebral bone-marrow changes in degenerative lumbar disc disease. An MRI study of 74 patients with low back pain. *The Journal of Bone and Joint Surgery. British volume*.

[21] Rahme R., Moussa R. (2008). The Modic vertebral endplate and marrow changes: pathologic significance and relation to low back pain and segmental instability of the lumbar spine. *American Journal of Neuroradiology*.

[22] Kulig K., Powers C. M., Landel R. F. (2007). Segmental lumbar mobility in individuals with low back pain: in vivo assessment during manual and self-imposed motion using dynamic MRI. *BMC Musculoskeletal Disorders*.

[23] Mitra D., Cassar-Pullicino V. N., McCall I. W. (2004). Longitudinal study of vertebral type-1 end-plate changes on MR of the lumbar spine. *European Radiology*.

[24] Hutton M. J., Bayer J. H., Powell J. M. (2011). Modic vertebral body changes: the natural history as assessed by consecutive magnetic resonance imaging. *Spine*.

